# Development of Norrin-Based Protein Therapeutic for Activation of Norrin-Wnt Signaling in Human Retinal Endothelial Cells

**DOI:** 10.3390/ijms262311340

**Published:** 2025-11-24

**Authors:** Kenneth P. Mitton, Wendy A. Dailey, Steven Q. Krikor, Kimberly A. Drenser

**Affiliations:** 1 Eye Research Institute, Oakland University, Rochester, MI 48309, USA; stevenkrikor@oakland.edu; 2 RetiNova Therapeutics, San Diego, CA 94402, USA; wdailey@retinovatherapeutics.com; 3 Associated Retinal Consultants P.C., Royal Oak, MI 48037, USA

**Keywords:** Norrin, Wnt signaling, blood retinal barrier, retinal vascular disease, endothelial cells, protein therapeutic, therapeutic development, recombinant protein

## Abstract

Norrin–Wnt signaling is essential for retinal vascular development and generation of the inner blood retinal barrier. Norrin itself is a potential therapeutic for retinal vascular repair. We explored the feasibility of producing a recombinant protein therapeutic based on human Norrin for intravitreal injection. Norrin^K86P^ production was tested using MBP fusion and non-tagged versions. FZD4 binding was evaluated by an ELISA, and the activation of *AXIN2* gene expression in primary human retinal microvascular endothelial cells was measured by qPCR. Intravitreal injection was tested in the rat eye, evaluated by fluoresceine angiography, OCT, and ERG. MBP-tagged Norrin was resistant to HRV3C protease cleavage unless linker polypeptides were also incorporated. MBP–Norrin or cleaved MBP–Norrin also required refolding with disulfide reshuffling to generate FZD4-binding activity and to affect *AXIN-2* gene expression. A production strategy based upon untagged Norrin^K86P^ refolded from bacterial inclusion bodies was selected. Intravitreal injection of Norrin^K86P^ did not affect retinal thickness nor retinal function, the latter monitored by the ERG A-wave and B-wave amplitudes. We concluded that MBP–Norrin, cleaved Norrin, and untagged Norrin from inclusion bodies display Norrin-like biological activity after refolding with disulfide reshuffling. The untagged, bacterial inclusion body process was selected for future large-scale bacterial fermentation. Norrin^K86P^ could be produced with Norrin-like biochemical and biological activities and was tolerated after intravitreal injection into the rat eye.

## 1. Introduction

Norrin (NDP, Norrie Disease Protein) is an atypical Wnt-protein that is essential for growth of the retinal vasculature and maintenance of the endothelial blood–retinal barrier [1]. Norrin’s presence as an endogenous retinal growth factor and its ability to improve neovascular regrowth in the mouse OIR model highlights Norrin itself as a candidate protein therapeutic for the repair and regeneration of a compromised BRB in retinal vascular diseases. This led us to explore the development of a bacterially produced version of human Norrin^K86P^ and report here that it displays Norrin activity in both binding to FZD4 and as an activator of Norrin target gene expression in primary human retinal microvascular endothelial cells.

Norrin is produced by Müller glial cells in the retina, and it binds to the Wnt receptor Frizzled-4 (FZD4) on endothelial cells [2]. Norrin functions as an angiogenic factor and as a neuroprotective growth factor [3], mediating angiogenesis partly through the induction of insulin-like growth factor-1 [4]. Frizzled-4 (FZD4) is the central receptor for Norrin. Early conditional knockout of Fzd4 in mouse endothelial cells impairs retinal vascular development while conditional knockout in adulthood causes conversion of endothelial cells from a PLVAP^(−)^ to PLVAP^(+)^ phenotype [5]. Unlike fenestrated endothelium, Plasma-Lemma Vesicle-Associated Protein (PLVAP) concentrations are very low in high-barrier endothelia of the CNS, including the neural retina. The essential role of Norrin to human retinal vasculature development was revealed by *NDP* gene variants that result in Norrie Disease or FEVR (Familial Exudative Vitreoretinopathy) [6,7,8,9]. Pathologic variants of Norrin are associated with several related vascular retinopathies, including persistent fetal vasculature syndrome (PFVS), retinopathy of prematurity (ROP), and Coats disease.

Norrin–Wnt signaling is essential for the formation of the three microvascular beds of the neural retina and for maintaining the high-barrier nature of the mature neural retina endothelium [1,10]. Norrin is secreted by Müller glial cells and binds to FZD4, triggering canonical Wnt signaling that regulates angiogenesis in the retina and the inner ear [11,12]. In the neural retina, Norrin stimulates the proliferation of the superficial vascular plexus and is important for the recruitment of Mural cells [13]. TSPAN-12 and LRP-5 are essential co-receptors that enhance this binding, and expression of this triple-receptor complex is a specific marker of neuroretinal endothelial cells. Retinal vascular defects that arise from the disruption of Norrin/FZD4 signaling can be mitigated by stabilizing beta-catenin. Conversely, inhibiting beta-catenin-dependent transcription results in vascular defects comparable to those observed with the inactivation of Norrin or its receptor components [14,15].

Transgenic expression of Norrin in NDP-deficient mice, ectopically from the lens, restores vascular development in the neural retina [16]. NDP^y/−^ transgenic mice with ectopic expression of Norrin from the retinal pigment epithelium also display accelerated vascular regrowth in a mouse model of oxygen induced retinopathy (OIR) [17]. We have demonstrated that a single injection of recombinant human Norrin protein can also accelerate vascular regrowth in the mouse OIR model [18]. More recently a tetravalent antibody to FZD4 and LRP5 was used to restore retinal angiogenesis and barrier function in Tspan12^−/−^ mice [19]. These results suggest that activation of the Norrin–Wnt signaling pathway is a therapeutic target for the repair of a compromised neuroretinal vasculature.

Norrin is a small, disulfide-rich protein (11 cysteines), with essential disulfide bonds for the tertiary structure of the monomer and for intermolecular stability of the biologically active dimer [12]. Norrin has been difficult to purify at a higher concentration from mammalian sources [20] or insect cells [21]. Expression of Norrin in *E. coli* results in inclusion body formation, and X-ray crystallography analysis of Norrin required the bacterial production of Norrin fused to the C-terminus of MBP (Maltose-Binding Protein) for solubility and isolation [12]. MBP-Norrin also required complete refolding to obtain active Norrin dimer formation. With this history in mind, seeking strategies to produce Norrin protein using bacterial production led us to investigate several options. While developed around Norrin protein, the results described here provide an example of strategies that can be applied to the production of other disulfide-rich mammalian proteins.

About one-third of mammalian proteins are synthesized in the endoplasmic reticulum (ER) for proper folding into their tertiary structures [22]. The ER compartment is more oxidizing than the cytoplasm, permitting the shuffling of disulfide bonds until native disulfide bonds are formed. Key signaling proteins, such as insulin and human growth hormones, need structural disulfide bonds for normal activity. Producing polypeptides in bacteria may not yield soluble active protein, but it generates large amounts of recombinant protein for purification. Recombinantly expressed proteins can be unfolded and refolded, if necessary, but refolding must avoid trapping the protein with the wrong disulfide bonds. This requires conditions that permit disulfide bonds to form reversibly and prevent aggregation of misfolded protein until the desired active protein is formed [23].

The first strategy we explored was the production of an MPB–Norrin fusion protein, the strategy used for previous X-ray structural studies [12], with an HRV3C protease-cleavable site between MBP and Norrin. We used SHuffle strains of *E. coli*, which have deletions of the genes for glutaredoxin reductase (Δgor) and thioredoxin reductase (ΔtrxB), permitting the formation of disulfide bonds in the cytoplasm. These strains also express a cytoplasmic version of the DsbC protein to facilitate shuffling of disulfide bonds [24]. DsbC exists as a dimer, has disulfide isomerase activity, and has a V-shaped cleft with protein chaperone activity. DsbC can bind misfolded proteins and catalyze the exchange of disulfide bonds until soluble folded structures are formed [25]. There is potential to produce mammalian proteins folded with their desired native disulfide bonds, although the chances of obtaining the desired native configuration may be reduced with increasing numbers of disulfides. Norrin is a small protein with seven essential disulfide bonds to form its active dimer.

We found that this soluble MPB–Norrin fusion protein was not protease-cleavable. A second version was then engineered, adding rigid alpha-helical spacers and a flexible linker [26] around the protease cut site, which was efficiently cleavable by the HRV3C protease. Both MBP–Norrin^K86P^ and Norrin^K86P^ were soluble but not active until refolded while supporting disulfide shuffling in vitro. This led to a final strategy to make tag-free Norrin^K86P^ proteins as insoluble inclusion bodies. Denaturing solvation and refolding with disulfide reshuffling in vitro resulted in protein with the biological activity of Norrin based on FZD4 binding and effects on primary human retinal microvascular endothelial cells (HRMECs).

## 2. Results

### 2.1. Expression of MBP-Norrin^K86P^ in E. coli SHuffle Strains

**Figure 1:**
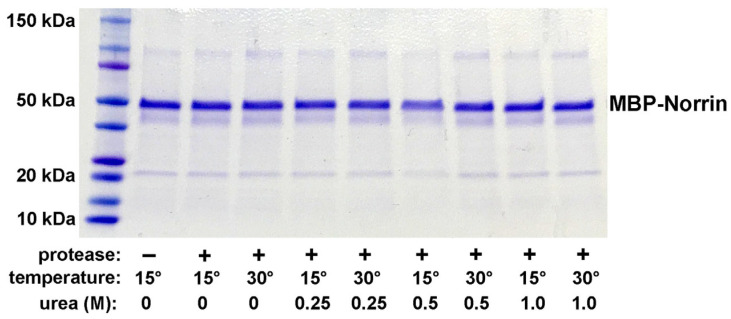
HRV3C protease cleavage testing of MBP–Norin in non-denaturing and denaturing conditions. Reactions were incubated for 18 h at 15 °C or 30 °C with 0, 0.25. 0.5, or 1.0 M urea with HRV3C viral protease (2 ng/µL). No evidence of cleavage was noted.

We first explored the potential expression of Norrin with disulfide bond formation as an N-terminal fusion protein with MBP. An MBP–Norrin fusion protein was previously utilized for X-ray crystallographic studies of a Norrin/FZD4 complex [12]. MBP fusion proteins are commonly used to promote solubility and for one step affinity capture and purification using resin with elution by maltose addition. Induction of protein expression with IPTG worked with both cell strains tested: SHuffle-T7 and SHuffle-T7 Express. After affinity isolation of MBP–Norrin with amylose resin, and elution with maltose, a strong Coomassie-stained protein band of the expected molecular weight was detected on DISC-PAGE gels. The SHuffle-T7 Express strain was taken forward for small-scale protein production. Cleavage of the MBP–Norrin with the HRV3C viral protease was found to be inefficient, tested over several hours, at low and higher temperatures, with and without urea for partial denaturation. No substantial release of Norrin was noted, as seen in Figure 1. 

**Figure 2:**
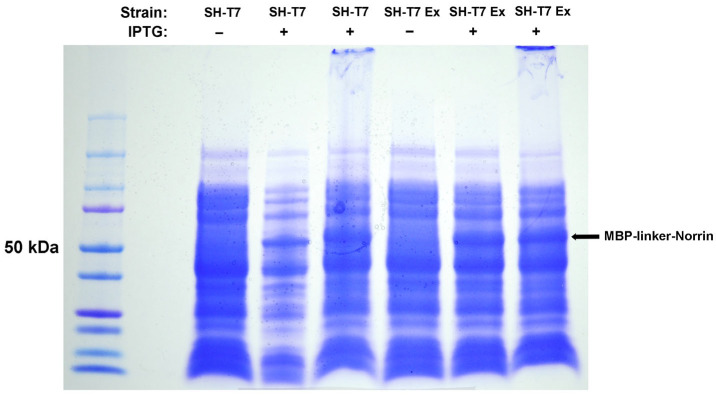
Expression of MBP-linker-Norrin in SHuffle-T7 and SHuffle-T7 Express strains of *E. coli*. DISC–PAGE gel of total protein extract after IPTG induction of MBP-linker-Norrin expression. SHT7-Express was selected as providing good recombinant protein production.

We concluded that the failure of HRV3C protease cleavage in the direct MBP–Norrin fusion construct was likely due to steric hindrance. This led us to modify the initial protein construct to a version-2, MBP-linker-Norrin, by engineering rigid and flexible linker polypeptide extensions around the viral protease cleavage site, as described in the methods. MBP-linker-Norrin expression (50 kDa) was also induced efficiently by IPTG in both SHuffle-T7 bacterial strains tested. The SHuffle-T7 Express strain was selected. See Figure 2.

**Figure 3:**
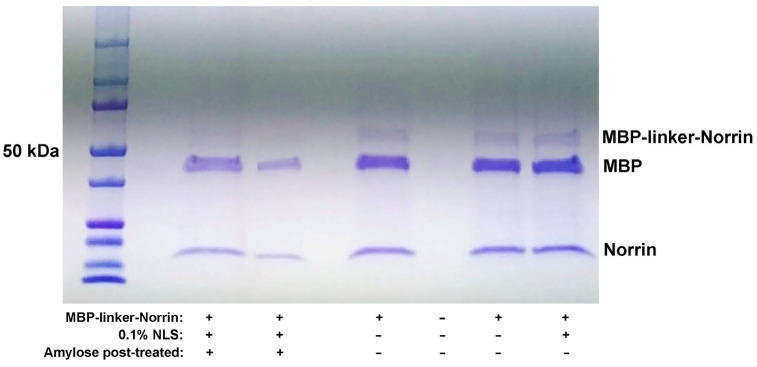
HRV3C protease cleavage testing of MBP-linker-Norrin. MBP-linker-Norrin was incubated with HRV3C protease for 4 h at 21 °C. Efficient cleavage of the fusion protein (middle lane) was observed with presence of a Norrin band. Proteins remained soluble at 4 °C over 24 h without or with addition of 0.1% N-lauryl-sarcosine (right lanes). A post-digest treatment with amylose resin reduced MBP content relative to Norrin (left lanes).

Standard isolation of soluble MBP-linker-Norrin with amylose resin was employed, and this version of the fusion protein was also tested for cleavage with the HRV3C protease. Addition of the linker extensions to separate the MBP and Norrin sub-regions resulted in an efficiently cleavable construct. (See Figure 3). Appearance of a Norrin construct at the expected lower molecular weight was concurrent with the disappearance of the MBP-linker-Norrin band.

### 2.2. MBP-Norrin: Frizzed-4 Receptor Binding

With the ability to cleave MBP–Norrin, both protease-cleaved MBP–Norrin and intact MBP–Norrin were tested for Frizzled-4 (FZD4)-binding activity using a receptor-binding ELISA plate assay. These proteins were first tested for binding without any attempt to refold soluble MBP–Norrin as extracted from bacterial preparation. Results indicated that both intact and protease-cleaved MBP-Norrin had lower than desired FZD4 binding activity. Protease cleaved preparation had more binding activity than uncut preparation but was still only 14% compared to a comparison control (R&D Systems human Norrin) (See Table 1). Both preparations were then processed for protein refolding with disulfide reshuffling. This process used 6M guanidine–HCl and 5 mM DTT to completely denature the proteins, followed by dialysis to remove guanidine–HCl in the presence of reduced and oxidized glutathione (GSH, GSSG) and 1 M arginine. Retesting the uncut and protease-cleaved preparations after this refolding process resulted in recovery of the expected FZD4 binding activity (see Table 1).

### 2.3. HRMEC Gene Expression with Refolded Norrin and MBP-Norrin

**Figure 4:**
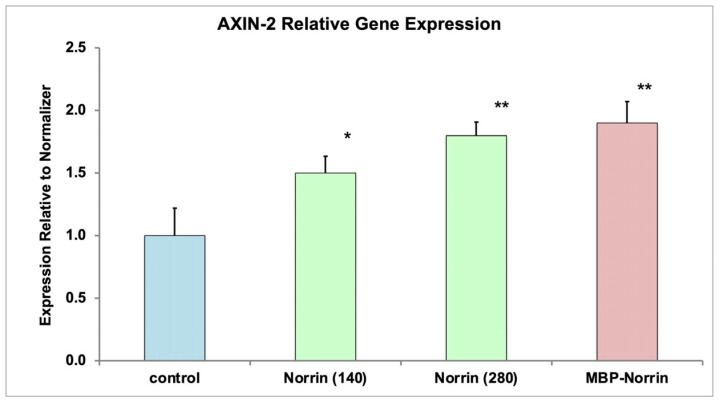
*AXIN-2* gene expression in primary HRMECs. Quantitative PCR analysis of *AXIN-2* gene expression in primary human retinal microvascular endothelial cells. Fractions of isolated Norrin and uncut MBP–Norrin, both after refolding with disulfide reshuffling, increased *AXIN-2* gene expression. Two concentrations of the refolded Norrin (140, 280 ng/mL) suggested a positive dose response. (ANOVA: * *p* < 0.05, ** *p* < 0.01, relative to control).

Refolded Norrin and refolded MBP–Norrin fractions were tested for activation of Norrin target gene expression in primary human retinal microvascular endothelial cells (HRMECs). *AXIN-2* gene expression was measured by qPCR analysis, 24 h after treatment of cells with Norrin or MBP–Norrin (see Figure 4). Both refolded Norrin and MBP–Norrin increased *AXIN-2* gene expression.

### 2.4. Expression of Tag-Less Norrin^K86P^ in E. coli

**Figure 5:**
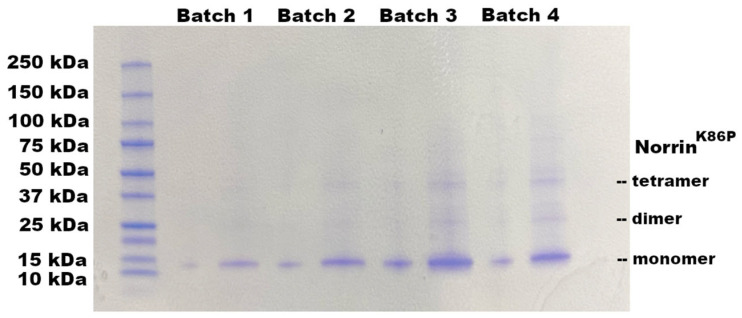
Expression of tag-less Norrin^K86P^ in BL21(DE3) cell inclusion body protein fractions. DISC-PAGE protein gel of inclusion body (IB) preparations from four separate batches are shown. IB material was dissolved and reduced by heating (100 °C × 10 min) in SDS sample buffer with 2-mercaptoethanol. Some dimeric and tetrameric bands can be formed during this treatment. Two different loading volumes (1 µL and 5 µL) were used for each of the four batches shown. Gels were stained with colloidal Coomassie blue. IB protein content was >98% recombinant Norrin^K86P^.

The requirement to refold bacterially produced Norrin, with disulfide reshuffling, suggested that Norrin could be made directly in *E. coli* as inclusion bodies (IBs) without any tags or fusion proteins. Norrin and other human proteins produced this way are highly concentrated as insoluble misfolded aggregates. However, this material provided an excellent first-stage purification process with high protein yield. A tag-less version of Norrin was then produced in *E. coli* BL21(DE3) cells. Bacterial inclusion body protein was isolated by lysis and extensive washing of the insoluble IB protein with Bug-Buster reagent. A highly enriched preparation of tag-less Norrin was obtained from the IB fraction, as shown in Figure 5, for four different batches of IB protein. The IB protein fraction was typically >95% recombinant Norrin.

### 2.5. Rat Retinal Vasculature and Retina Thickness After Norrin^K86P^ Intraocular Injection

Rats received intraocular injections (2.5 µL), vehicle in the OD eye, and 250 ng Norrin^K86P^ in the OS eye. Fluorescein angiography (FA) of the retinal vasculature and SD-OCT imaging for retinal thickness was used to compare the tolerance of the retina for intravitreal injections. Fundus images, FA images, and SD-OCT analysis compared all test eyes pre-injection to 3 weeks post-injection.

**Figure 6:**
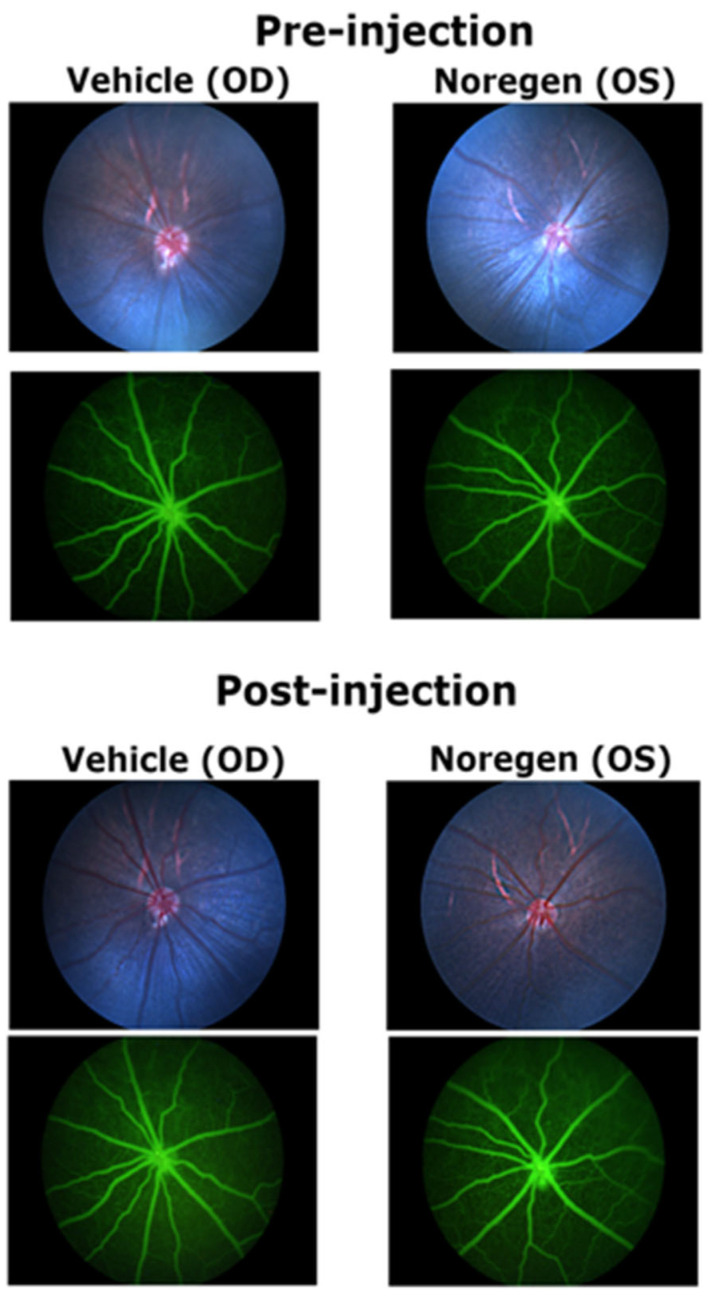
Fundus and fluorescein angiography images before and after injection of Norrin^K86P^. Long Evans rats were imaged pre injection and 3 weeks post injection of 250 ng Norrin^K86P^. An example of the same Norin^K86P^-treated eye (OS) and vehicle-treated contralateral eye (OD) are shown. Brightfield fundus images are shown for each eye with their corresponding fluoresceine angiography image below. Angiography shows the perfused microvasculature of the neural retina (green). No impact on the retinal vasculature was noted for any eyes, whether Norin^K86P^- or vehicle-injected, across all rats tested. (N = 4). The same retinas were also analyzed for retinal thickness using SD-OCT.

Fundus images and FA images did not reveal any impact on the retinal vasculature when comparing before and after injection for any of the eyes, whether vehicle- or Norrin^K86P^-injected. This was the case for four different rats. Figure 6 shows an example of pre-injection and post-injection fundus and FA images. The primary retinal vessels remained normal in appearance with no evidence of dilation in vehicle-injected and 250 ng Norrin^K86P^-injected eyes. Primary veins and arteries remained identifiable with a normal morphology: primary retinal veins had a larger diameter than primary arteries, with a distribution alternating around the optic disk (Figure 6).

**Figure 7:**
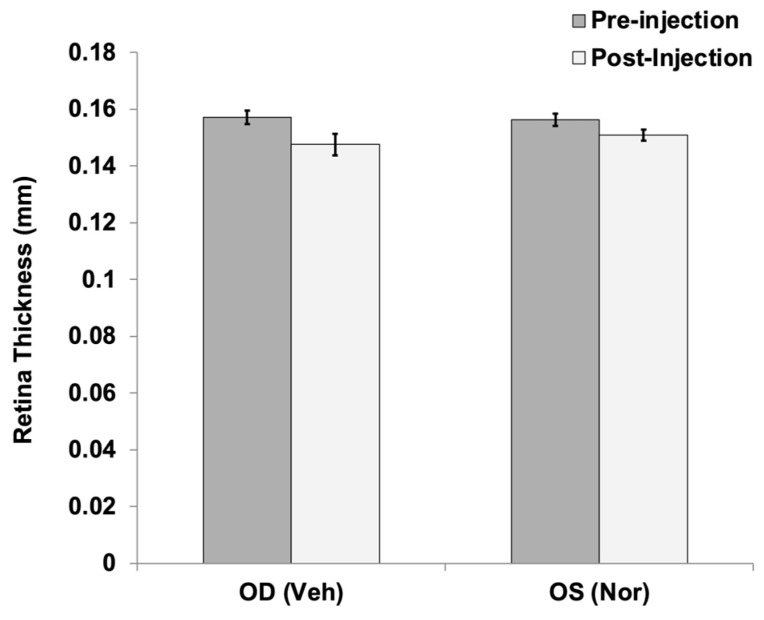
Retinal thickness before and after intraocular injection of Norrin^K86P^. Long Evans rats received intraocular injections of vehicle (Veh) OD and 250 ng of Norrin^K86P^ (Nor) OS. Average retinal thickness was calculated from 24 measurements around the optic disk for each retina, before injection and then again three weeks post injection. Thickness was measured between the inner limiting membrane (ILM) and outer limiting membrane (OLM) of the neural retina. The retinal thickness values (mm) averaged from four different rats are shown; bars show standard deviation.

In addition to fundus and FA imaging, the retinal thickness of Long Evans rats injected with 250 ng Norrin^K86P^ was analyzed in vivo using SD-OCT, and Diver 2.0 software (Bioptigen). No appreciable differences in retinal thickness were noted comparing vehicle-injected (OD) to Norrin-injected (OS) eyes before and after intraocular injection, as graphed in Figure 7.

### 2.6. Rat Photoreceptor Response (ERG) In Vivo After Norrin^K86P^ Intraocular Injection

**Figure 8:**
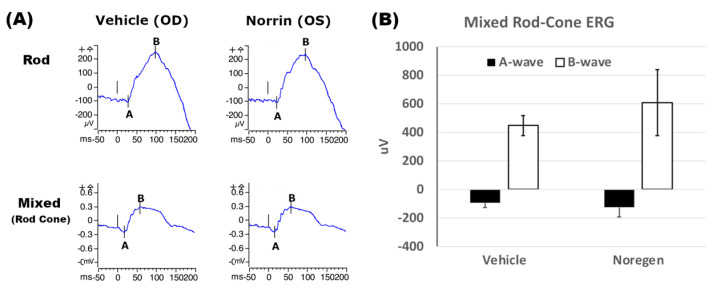
Mixed Rod–Cone ERG response of Long Evans rats. Long Evans rats received intraocular injections of vehicle (Veh) OD and 250 ng of Norrin^K86P^ (Nor) OS. (**A**) Examples of Rod and mixed Rod–Cone ERG recordings from vehicle and contralateral Norrin^K86P^-injected eyes, 3 weeks post-injection. The left vertical marker indicates the light pulse flash at 0 milliseconds (ms). (**B**) Mixed Rod–Cone ERGs were measured, comparing vehicle-injected (OD) and Norrin-injected eyes, after dark adaptation. Average magnitudes of A-wave and B-wave (uV) are shown, using full-field white-light stimulation of 3.0 cd-sec/m^2^. Bars show standard deviation (N = 4 rats). No inhibition of Cone–Rod ERG response was detected in Norrin-treated versus vehicle-treated eyes.

Functional testing for any effects of Norrin^K86P^ treatment (250 ng intravitreal injection) on retinal function was evaluated using full-field ERG of Long Evans rats, 3 weeks post-injection. Examples of ERG traces for Rod-only (dim flash) and mixed Rod–Cone (bright flash) responses in dark adapted rats are shown in Figure 8A. Rod and Rod–Cone ERG responses were of similar appearance between vehicle-injected eyes and Norrin-injected eyes. The average A-wave and B-wave amplitudes were similar when comparing vehicle-injected and Norrin-injected eyes. See Figure 8B. No inhibition of ERG response was noted from Norrin treatment.

## 3. Discussion

Initial development of bacterial recombinant Norrin protein expression first utilized MBP (Maltose-Binding Protein) fused to the N-terminal of the mature human Norrin amino acid sequence. This was explored because it is known that MBP–Norrin can be produced in *E. coli,* and in fact, this strategy was utilized by Ke Et Al. for X-ray crystallographic analysis of Norrin dimers bound to a Frizzled-4 and LRP5/6 complex [12]. As illustrated in our results (Figure 1), we found that purified MBP–Norrin could not be cleaved by the HRV3C viral protease, even though a cleavage site was provided between the MBP and Norrin moieties. While potentially useful for NDP dimerization studies, we desired to produce a recombinant Norrin protein without the large MBP tag.

Based on our hypothesis that the MBP and Norrin structures were too large to allow access of the HRV3C protease to its cleavage site (LEVLFQGP), we tested a second construct, engineered to add rigid helical linker domains and additional flexible linkers surrounding the protease cleavage site. The intent was to increase the separation and flexibility between the MBP and Norrin components to improve protease access. Indeed, this strategy worked, and the MBP-linker-Norrin was a very efficient substrate for the HRV3C protease.

Aside from our particular interest in Norrin, this result suggests a useful strategy that could be employed when faced with fusion protein constructs that suffer a similar lack of physical access to an intermediate protease cleavage site. The amino acid sequence (EAAAK) has been used previously to add rigid helical linkers, while the glycine-rich sequence (GGGGS) provides the addition of flexible linkers [26]. Engineering the addition of these linkers provided a viable solution to allow for protease cleavage of MBP from Norrin.

After solving a way to cleave recombinant Norrin from MBP, it was discovered that the resulting Norrin, while soluble, did not display the expected activity for the binding to Frizzled-4 in plate-binding assays. Expression in T7-Shuffle *E. coli* strains to permit disulfide bond formation resulted in Norrin that was soluble but lacked the required binding activity. We hypothesized that this initial Norrin was soluble, but only a small percentage of the Norrin was correctly folded with the required disulfide bonds. Thus, only a small percentage of this Norrin was active for Frizzled-4 binding. This directed us to a strategy of disulfide shuffling to refold Norrin into its native conformation while preventing the protein from becoming trapped in permanent but incorrect disulfide bonds.

Norrin is a disulfide-rich protein with a high density of cysteines, creating opportunity for incorrect disulfide bond pairings. We next employed complete denaturation and a refolding process that included disulfide reshuffling. This resulted in the recovery of Frizzled-4-binding activity. Refolding of either intact or cleaved MBP-linker-Norrin resulted in an increase in Norrin-like Frizzed-4-binding activity. This result established the requirement to denature and refold Norrin while permitting disulfide reshuffling. Based upon this conclusion, it was decided to switch to a third and final strategy in which tag-less Norrin was expressed in *E. coli* as inclusion bodies, which would be mostly recombinant Norrin.

Expression of Norrin^K86P^ devoid of additional amino acid tags resulted in the expected production of a substantial amount of Norrin protein in the insoluble inclusion body fraction from bacterial cultures. While inclusion bodies of recombinant proteins are crystalline, insoluble, and generally comprise misfolded protein, they provide a substantial source of recombinant protein that is 95–99% recombinant protein. Inclusion body proteins can be utilized by first solvating them in protein denaturing conditions and then refolding the proteins [23]. In the case of Norrin, refolding had to occur in conditions that permitted disulfide reshuffling. In that process, disulfide bonds are allowed to reversibly form, so the protein does not become trapped in a non-functional conformation by the wrong disulfide bond pairings. This requires a denaturing agent, such as 6M guanidine–HCl, to first solvate the IB protein. Refolding then involved a gradual reduction in the guanidine–HCl concentration by dialysis, in the presence of a mixture of both reduced (GSH) and oxidized glutathione (GSSH). In addition, a high concentration of arginine was used to prevent aggregation of intermediate misfolded protein.

The refolding process of inclusion body protein was successful for generating recombinant Norrin^K86P^ that was biologically active. Norrin biological activity was evaluated in two ways. First, using in vitro assays of Frizzled-4 binding, refolded Norrin^K86P^ preparations displayed Frizzled-4-binding activity, compared to a control Norrin preparation. Second, refolded protein increased expression of the *AXIN-2* gene in primary HRMECs, a known effect of Norrin.

Preparation of proteins like Norrin in bacteria requires sufficient purification from bacterial cell wall glycoproteins to allow introduction of the recombinant protein into the ocular environment. For the purposes of basic laboratory process development, we employed the strategy of denaturing size exclusion chromatography. That is, after denaturing and solvating inclusion body protein, the denatured recombinant Norrin was separated further from bacterial proteins under denaturing conditions (guanidine–HCl) prior to refolding the protein with disulfide reshuffling. This kind of preparation was used for testing biological activity but also tested with intraocular injection into rat vitreous to test ocular tolerance.

Analysis of rat retinas after injection of a large dose of Norrin^K86P^ (250 ng, into vitreous) were completed using fluorescein angiography and SD-OCT analysis of retinal thickness. No changes to the retinal vasculature were detected when comparing fluorescein angiography images of vehicle-injected eyes and contralateral Norrin-injected eyes, before and after Norrin injection. Likewise, SD-OCT measurements of neural retina thickness did not find any significant differences when comparing vehicle- and Norrin-injected eyes. Finally, ERG analysis of retinal function did not reveal any ill effects of Norrin injection on either the Rod-only or mixed Rod–Cone ERG response. We concluded that this modest purification process of recombinant Norrin resulted in sufficiently low concentrations of bacterial protein to permit introduction into the living rodent eye.

### Summary

To demonstrate feasibility, a process was established to produce a recombinant protein based on the human Norrin protein in bacterial culture. Recombinant Norrin protein has the potential as a future therapeutic for the repair of a damaged retinal vasculature and blood–retinal barrier due to Norrin’s biological function. Norrin is active as a dimer, is disulfide-rich, and has proven to be difficult to produce recombinantly. Our initial explorations of MBP-tagged strategies resulted in demonstrating the use of protein linker additions to solve the previous challenge of steric access for HRV3C protease cleavage. The strategy described here may be useful for similar situations with different fusion protein constructs. However, the need to completely refold recombinant protein to obtain Norrin’s biological activity led to a final production strategy based on expressing untagged Norrin^K86P^ protein as bacterial inclusion bodies (IBs). The best final process was found to be denaturation solvation of IB protein followed by denaturing SEC and refolding of the protein using disulfide reshuffling conditions. We concluded that it was feasible to produce biologically active recombinant Norrin^K86P^ in substantial quantity using bacterial fermentation and that the preparations were well tolerated by the eye after injection into the vitreous of Long Evans rats.

## 4. Materials and Methods

### 4.1. Animal Care and Use

All animal care and tissue collections performed in this study were carried out with the approval of Oakland University’s Institutional Animal Care and Use Committee, and research was carried out in compliance with the requirements contained in the ARVO Statement for the Use of Animals in Ophthalmic and Vision Research (Association for Research in Vision and Ophthalmology, www.ARVO.org), IACUC approval number 2021–1130. Adult Long Evans rats (155–190 g body weight, female) were obtained from Charles River Laboratories (Wilmington, MA, USA) and were housed at Oakland University in a facility approved by the Association for Assessment and Accreditation of Laboratory Animal Care International (AAALAC).

### 4.2. Bacterial Protein Expression Plasmids 

**Figure 9:**
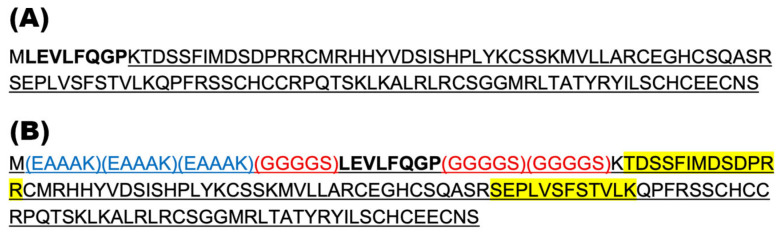
Norrin-based protein sequences inserted into the pD454-MBP vector. Inserted sequences were added in frame following the MBP sequence. The resulting proteins were MBP N-terminal fusion proteins. (**A**) Nor-2 contained the mature human Norrin^K86P^, sequence underlined. The HRV3C protease cleavage site is shown in bold font. (**B**) Nor-w-Linker included extra linker sequences to improve the steric exposure of the HRV3C cleavage site. Three rigid helical linkers (blue font) and three flexible linkers (red font) are indicated. Two polypeptides identified by mass spectroscopy after electrophoresis of a Norrin-sized band (15 kDa) obtained after HRV3C protease treatment are highlighted with yellow.

Plasmids for bacterial protein expression were designed, synthesized, and inserted into the pD454-MBP or pD454 plasmids (Atum, https://www.atum.bio/, Newark, CA, USA). The pD454-MBP plasmid provided for inducible expression, in *E. coli*, of an N-terminal fusion of mature Norrin^K86P^ with the Maltose-Binding Protein (MBP). An HRV3C protease target site was present between the Norrin and MBP domains (See Figure 9). The pD454 plasmid provided for the inducible expression of mature human Norrin^K86P^ without the MPB fusion or any additional tags. The protein encoding sequences were configured to use bacteria-prefered codons in *E. coli*. The plasmid construct Nor-2 was a direct fusion of MBP with human Norrin. The plasmid construct Nor-w-Linker was similar but added extra polypeptide linker sections, positioned on either side of the HRV3C protease cleavage site (Figure 9). The plasmid construct pD454-Nor encoded only mature human Norrin^K86P^ without N- or C-terminal tags.

### 4.3. Bacterial Strains for Recombinant Protein Expression

SHuffle T7 (SHT7), SHuffle T7 Express (SHT7-Express), and BL21(DE3) competent *E. coli* cells (New England BioLabs, Ipswich, MA, USA) were used for bacterial protein expression. BL21(DE3) was used for the expression of untagged Norrin as bacterial inclusion body protein.

### 4.4. Transformation of Bacterial Strains for Norrin^K86P^ Protein Expression

Plasmid DNAs were diluted in TE pH 8.0 to a working concentration of 0.1 ng/µL for transformation. In total, 2 µL of plasmid DNA was mixed into a freshly thawed aliquot of competent SHT7 or 1 µL of DNA with competent SHT7-Express cells or 3 µL for BL21(DE3) cells. Tubes were flicked 5 times to mix and incubated on ice for 30 min. DNA bacterial mixes were then subjected to 42 °C heat shock for 30 s for the SHT7 strains or 10 s for BL21(DE3), in a water bath, and then incubated on ice for 5 min. SOC media (950 µL) was added to each transformation mix; these were incubated for 60 min at 250 RPM and 30 °C. Transformation mixtures were centrifuged for 2 min 5000× *g* to pellet bacteria and the supernatant was discarded. Cells were resuspended in SOC media (100 µL). To select transformed clones, by ampicillin resistance, 5 µL and 95 µL of the cells were spread onto prewarmed LB/AMP plates (100 µg/mL ampicillin) and incubated for approximately 10 min to dry. Plates were then incubated overnight at 30 °C to allow individual colonies to form. Several clonal isolates of each transformation were used for the preparation of glycerol stocks, combining 850 µL of the overnight culture and 150 µL of glycerol (15%), followed by incubation on ice for 10 min, and then transfer to −70 °C.

### 4.5. Extraction of Inclusion Body Protein from E. coli

Extraction of inclusion body protein from *E. coli* was possible using common treatment with lysozyme and sonication, but more efficient extraction of inclusion body (IB) protein was afforded using a mixture of two recombinant enzymes, Benzonase and rLysosyme (BugBuster MasterMix (BBMM), Sigma Aldrich, St. Louis, MO, USA). This process degraded the bacterial cell wall, DNA and RNA, to allow more efficient washing of IB protein. Using the manufacturer’s instructions, 0.5 g of frozen bacterial cells were resuspended in 2.5 mL of BBMM solution, supplemented with 0.5 mM PMSF (phenylmethylsulfonyl fluoride, serine protease inhibitor) and incubated at room temperature for 15 min. An equal volume of BBMM was then added for an additional 10 min of incubation. After dilution with 9 volumes of deionized water (to 10% BBMM), inclusion body protein was collected by centrifuging (3200× *g* 25 min, 5 °C). IB material was resuspended and washed two times with 5 mL of 0.1× BBMM, and then collected by the centrifuge (3200× *g* 20 min, 5 °C). A final third wash in 1 mL of 0.1× BBMM was completed, with the final IB protein pellet collected in a microcentrifuge (20,000× *g* 15 min, 5 °C). The IB material was weighed and frozen.

### 4.6. Denatured SEC for Inclusion Body Protein

IB protein was solvated using denaturation conditions and then fractionated using size exclusion chromatography under denaturing conditions (Denaturing SEC). The IB protein was gently suspended and dissolved in unfolding buffer (10 mg/mL IB, 49 °C, 30 min): 50 mM Tris–HCl pH 8.5, 5 mM EDTA, 7 M guanidine–HCl, and 5 mM DTT. Solvated IB protein was centrifuged (1 min 20,000× *g*) to remove any insoluble material prior to column loading. The SEC column comprised Bio-Gel P100 Polyacrylamide Gel packed into a 100 mL bed volume Econo-column column per the manufacturer’s instructions (Bio-Rad, Hercules, CA, USA), equilibrated with a column running buffer (50 mM Tris–HCl pH 8.5, 4 M guanidine–HCl). The column was run using top shelf gravity feed pressure and 1 mL fractions collected using a Pharmacia Frac-100 fraction collector. Eluted protein was monitored by A280 analysis of each fraction. A total of 50 µL of selected fractions were ethanol precipitated, separated by DISC-PAGE, and processed with Imperial Protein Stain (Thermo Fisher, Waltham, MA, USA) to identify Norrin fractions.

### 4.7. Refolding of Recombinant Norrin^K86P^ with Disulfide Reshuffling

Refolding of Norrin^K86P^ required conditions to remove guanidine–HCl gradually while preventing aggregation of incompletely folded protein during the transition from the denatured state to secondary and tertiary structures. Another essential requirement was to provide conditions that permitted the dynamic formation of disulfide bonds, without trapping Norrin^K86P^ in an inactive tertiary structure. This was accomplished by dialysis of the denatured Norrin^K86P^ protein fraction in refolding buffer: 20 mM Tris pH 8.5, 1 mM EDTA, 1 M L-Arginine, 500 mM NaCl, 1 mM GSH, and 1 mM GSSG. Dialysis used 10 kDa cutoff dialysis cassettes (Thermo Fisher).

### 4.8. Anesthesia for Intraocular Injections, Retinal Imaging, or ERG Testing

Pupils were dilated with tropicamide and phenylephrine eye drops (Covetrus, Portland, ME, USA) prior to anesthesia. Rats were anesthetized with an intraperitoneal injection of a Ketamine HCL (50 mg/kg) and Xylazine (7 mg/kg) (Covetrus).

### 4.9. Norrin^K86P^ Intravitreal Injection (Rat)

Pupils were dilated with tropicamide and phenylephrine drops prior to anesthesia. Rats were anesthetized with an intraperitoneal injection of a Ketamine HCL (50 mg/kg) and Xylazine (7 mg/kg). Rat retinas were imaged before and 3 weeks after injections of 250 ng Norrin^K86P^, using fluorescein angiography and SD-OCT imaging to record the status of their normal retinal vasculature. Recombinant Norrin^K86P^ was diluted with phosphate-buffered saline solution (1× PBS, no calcium, no magnesium) to a concentration of 100 ng/µL for intravitreal injections of a 2.5 μL volume (dose 250 ng/eye). Injections were given with a NanoFil syringe with 35-gauge beveled NanoFil needles (WPI Inc., Sarasota, FL, USA). The contralateral eye served as a control and received the same injection procedure using vehicle alone (1× PBS).

### 4.10. Fluorescein Angiography (FA)

After sedation and pupil dilation, rats were injected (intraperitoneal) with 50 µL of 10% sodium fluorescein in 1× PBS. Corneal surfaces were protected with GenTeal lubricant eye gel (Novartis, CVS Pharmacy) to maintain corneal hydration and to provide optical transmission with the camera lens. Retinal blood vessels were imaged using a MICRON-3 camera system (Phoenix Micron, Bend, OR, USA), using normal white-light illumination fundus images and a dial-in fluorescein filter set for capturing FA images (blue illumination, green emission).

### 4.11. Analysis of Rat Retinal Thickness In Vivo

Spectral Domain Optical Coherence Tomography (SD-OCT) was employed to measure retina thickness in vivo, comparing before and 3 weeks after injection of 250 ng Norrin^K86P^. To maintain corneal transparency, artificial tear lubricant eye drop solution was applied often to both corneal surfaces. Anesthetized rats were secured in a three-axis positioning support cradle (Bioptigen, Durham, NC, USA). SD-OCT scans were taken using an Envisu R2200 model SD-OCT system (Bioptigen), equipped with a lens for the rat eye axial length. A rectangular scan pattern of rat sizes 2.6 mm × 2.6 mm was used (1000 A-scans by 100 B-scans). For measurement of retinal thickness, retinal layers were marked and measured using processed OCT images with InVivoVue Diver 2.0 software (Bioptigen), as previously described [27]. A fixed 5 × 5 grid was first centered on the optic disk. Boundaries of all retinal layers were marked at measurement grid locations. The central grid position was not used, as it marks the optic disk center. The remaining 24 grid positions were then used to generate an average thickness between the inner limiting membrane (ILM) and the outer limiting membrane (OLM) for each eye, both pre and post injection. Retinal thickness was measured between the ILM and the OLM.

### 4.12. Electroretinography (ERG)

ERG analysis was used to compare the Rod and mixed Rod–Cone responses of Long Evans rat retinas before and 6 weeks after intraocular injection of 250 ng Norrin^K86P^ in vivo. A Diagnosys LLC Espion-III ERG system with ColorDome was used to obtain the full-field ERG responses in response to white light flash stimulation. Rats were dark-adapted for 2 h and then handled under red light room lighting in a rodent ERG imaging suite. Pupils were dilated with two applications of tropicamide and phenylephrine–HCl eye drops to ensure maximum pupil size prior to anesthesia. Once anesthetized, rats were positioned on a custom support platform with an integrated warming pad to maintain normal body temperature. Gold loop electrodes were used for electrical coupling to corneas bathed in a small application of corneal protectant (GenTeal lubricant gel). One platinum microneedle was inserted into the hind flank skin (ground electrode), and a second platinum microneedle (reference electrode) was inserted into the skull cap skin just forward of the ears to minimize the detection of cardiac activity (EKG) in the ERG trace. A custom program was used to reproducibly automate the ERG testing. For the Rod-only response, dark-adapted rats were first stimulated with white light (6500K) flashes using a dimmer illumination intensity of 0.01 cd-s/m^2^. Immediately after the Rod test sequence, the mixed Rod–Cone ERG sequence was recorded using a brighter flash intensity of 3 cd-s/m^2^ to activate both Rod and Cone photoreceptors. Five traces were time-averaged to generate all ERG traces.

### 4.13. Receptor Binding ELISA

An In Vitro ELISA was created to assess Norrin^K86P^ potency by its ability to bind to its receptor, Frizzled-4 (FZD4). Briefly, a rhFrizzled-4/FC Chimera (R&D Systems, Minneapolis, MN, USA; 5847-FZ-050) was coated onto the surface of a 96-well plate overnight. The next day, the plate was washed with buffered surfactant (R&D Systems # WA126). The surfaces were then blocked with BSA (1% in PBS) or Casein (1% in PBS) and a serial dilution of the test Norrin^K86P^ (0.8–500 ng/mL) was made in the blocking agent. Each of the 7–9 levels of Norrin^K86P^ were added to duplicate or triplicate wells of the plate and allowed to incubate for 2 h. After washing away unbound Norrin^K86P^, a biotinylated Norrin antibody (R&D Systems; BAF3014) was added to the wells and allowed to incubate for 2 h at room temperature. Following incubation with the antibody, the wells were washed and incubated with Streptavidin–HRP (R&D Systems; DY998), followed by more washing. TMB substrate (R&D Systems, DY999) was added to the wells, the plate was stored in the dark, and color development was stopped by the addition of 2N Sulfuric Acid after 20 min. The absorbance at 450 nm was read on a BioTek Cytation 3 or Epoch 2 Plate Reader. The data was analyzed by four-parameter logistic (4-PL) curve fitting to determine the half maximal effective concentration (EC_50_).

### 4.14. Culturing of Primary Human Microvascular Retinal Endothelial Cells (HMREC)

Primary HMRECs were obtained from Cell Systems (Kirkland, WA, USA). They were grown to confluence in six well plates that had been pre-coated with Cell Attachment Factor (Cell Systems, Kirkland, WA, USA) and grown to confluence in fully supplemented EndoGRO-MV media (Millipore, Burlington, MA, USA), a low serum media that does not contain VEGF. Supplements included EndoGRO-LS supplement (0.2%), rh EGF (5 ng/mL), L-Glutamine (10 mM), Heparin Sulfate (0.75 U/mL), Ascorbic Acid, (50 µg/mL), FBS (5%), and Hydrocortisone Hemisuccinate (1 µg/mL). The cells were weaned to media containing no Hydrocortisone Hemisuccinate prior to stimulation with Norrin^K86P^ or *rh*Norrin (R&D Systems, Minneapolis, MN, USA; 3014-NR). Concentrations ranged from 2 to 2000 ng/mL, and after a 24 h duration, the cells were trypsinized and collected for RNA isolation.

### 4.15. Quantitative PCR Analysis of HMREC Gene Expression

Total RNA was isolated using the Monarch Total RNA Miniprep kit (NEB, Ipswich, MA, USA; T2010) with the optional On-Column DNase I treatment to remove residual DNA, according to the kit instructions. First-strand cDNA was synthesized by reverse transcribing 1 µg of total RNA per sample using the LunaScript RT Super Mix Kit (NEB, Ipswich, MA, USA; E3010L). The reaction conditions were according to the manufacturer’s instructions: 25 °C for 2 min, 55 °C for 10 min, and 95 °C for 1 min. All compared samples were processed using the same reagent set. Stock first-strand cDNA preparations were stored at −70 °C and were not used for analysis after a maximum of three freeze-thaws. qPCR was performed using a duplex reaction format with FAM-labeled probe/primer pairs for the gene of interest and VIC-labeled probe/primer-limited pairs for TBP (Tata-Binding Protein) as the normalizer gene. For real-time PCR reactions, sample first-strand cDNA was diluted 5-fold with deionized water, and 2 µL was added to 18 µL of master mix for 20 µL PCR reactions. Triplicate reactions were used for each sample using the Luna Universal Probe qPCR 2× Master Mix with Rox reference dye (NEB, Ipswich, MA, USA; M3004). Reactions were run on an AriaMx Real-time PCR System (Agilent, Santa Clara, CA, USA) and the comparative method used to determine the relative gene expression. Gene expression assays were evaluated for high PCR efficiency using a dilution series of HMREC cDNA to ensure validity using the delta–delta Ct method for comparing relative gene expression. Each replicate reaction was internally normalized relative to endogenous *TBP* gene expression. The specific assay probe sets used for gene expression analysis are listed in Table 2.

## Figures and Tables

**Table 1:** MBP–Norrin Frizzled-4-binding activity. Percent of expected potential binding activity relative to comparison control (R&D Systems Norrin).

Preparation	Before Refolding (%)	After Refolding (%)
Uncut	7	94
Cut	14	101

**Table 2:** Taqman gene expression probes for HRMEC gene expression.

Gene	Probe Set Index	Spans Exons
*AXIN2*	Hs00610344_m1, FAM	4–5
TBP	Hs00427620_m1, VIC-PL	3–5

## Data Availability

There are no raw data sets deposited in public systems related to the experiments presented here. Questions may be directed at the corresponding authors, and they will be handled on a case-by-case basis. Some information associated with the drug development process can only be released with the permission of RetiNova Therapeutics. All progress reports associated with this, and any NEI/NIH supported research, are available to the public via the current NIH access points.

## Abbreviations

The following abbreviations are used in this manuscript:

OCT	Optical Coherence Tomography
SD-OCT	Spectral Domain Optical Coherence Tomography
ERG	Electro Retina Gram
MBP	Maltose Binding Protein
IB	Inclusion Body
FZD4	Frizzled 4
